# Evaluation of a short instrument for measuring health-related quality of life in oncological patients in routine care (HELP-6): an observational study

**DOI:** 10.3389/fpsyg.2023.1158449

**Published:** 2023-05-16

**Authors:** Theresa Schrage, Mirja Görlach, Christian Stephan Betz, Carsten Bokemeyer, Nicolaus Kröger, Volkmar Mueller, Andreas Krüll, Holger Schulz, Christiane Bleich

**Affiliations:** ^1^Department of Medical Psychology, University Medical Center Hamburg Eppendorf, Hamburg, Germany; ^2^Department of Otolaryngology, University Medical Center Hamburg Eppendorf, Hamburg, Germany; ^3^II. Medical Clinic and Polyclinic, University Medical Center Hamburg Eppendorf, Hamburg, Germany; ^4^Department of Stem Cell Transplantation, University Medical Center Hamburg Eppendorf, Hamburg, Germany; ^5^Department of Gynecology, University Medical Center Hamburg Eppendorf, Hamburg, Germany; ^6^Department of Radiotherapy and Radiation Oncology, University Medical Center Hamburg Eppendorf, Hamburg, Germany

**Keywords:** health-related quality of life (HrQoL), psycho-oncology, psychometrics, oncological routine care, confirmatory factor analysis (CFA)

## Abstract

**Purpose:**

Patient-reported outcomes have not been sufficiently implemented into the routine care of cancer patients because the existing instruments are often too long and complex or not cancer-specific. The aim of this study is the determination of psychometric properties and item reduction of a newly developed health-related quality of life (HrQoL) questionnaire for use in oncological clinical routines.

**Methods:**

This observational study with a repeated measurements design included oncological inpatients and outpatients. A total of 630 patients participated at the first point of measurement and 404 at the second point of measurement. To evaluate the instrument, we conducted hierarchical confirmative factor analyses and for further validation correlated the resulting factors with standardized and validated HrQoL measurements. Test–retest reliability and responsiveness to change were tested.

**Results:**

The developed questionnaire “HELP-6” (“Hamburg Inventory for Measuring Quality of Life in Oncological Patients”) has a six-factor structure and has moderate-to-good convergent validity (*r*= −0.25 –−0.68). Test–retest reliability was moderate-to-good (*r* =0.56−0.81, *p* < 0.001). Indications for responsiveness to change were found for three dimensions. The final version of the questionnaire HELP-6 has six dimensions with one item each.

**Conclusion:**

With the HELP-6 instrument for measuring HrQoL in cancer patients, we provide a short and practical patient-reported outcome instrument. Though responsiveness to change could not be confirmed for all dimensions in this study, the HELP-6 includes time-efficient completion and evaluation and is informative in relevant HrQoL dimensions of cancer patients. Therefore, the HELP-6 poses an important addition to inpatient and outpatient routine cancer care.

**Trial registration:**

This study was registered at Open Science Framework (https://osf.io/y7xce/), on 9 June 2018.

## Introduction

The assessment of patient-reported outcomes (PROs) and health-related quality of life (HrQoL) has become an important part of cancer care (Van Egdom et al., 2019; Park et al., 2021; Toh et al., 2022). By introducing PROs, active collaboration and shared decision-making between patients and clinicians can be encouraged (Snyder et al., 2012; Schuler et al., 2017). Furthermore, the administration of oral cancer therapies is growing and with it fewer consultations with clinicians (Esper, 2013). Consequently, clinicians need more feedback on patients' HrQoL, such as patient-reported outcomes measurements (PROMs). PROs have not been sufficiently implemented in routine care because existing measurements are often too long and complex to evaluate, or they are not cancer-specific (Bascioni et al., 2005; Boyce et al., 2014).

Thus, for a continuously used instrument, factors such as less time required for completion, easy comprehensibility, and simple scoring and interpretation of the questionnaire are necessary (Lewis et al., 2019; Van Der Willik et al., 2019; Nguyen et al., 2020; Atallah et al., 2021). These features are important because many patients are too restricted to answer extensive questionnaires. Additionally, the questionnaires should be filled-out several times at shorter intervals to be able to use PROs for monitoring (Bascioni et al., 2005; Lewis et al., 2019; Atallah et al., 2021).

In routine care, an applied measurement needs to achieve a balance between accuracy, coverage of important HrQoL domains, and usefulness of the information in a time-consuming setting. Implementation research reveals several facilitators, as well as barriers to the implementation and broad use of PROMs in routine care. Barriers to routine care can be a lack of knowledge by healthcare professionals (HCP) on how to interpret and use PROM results during treatment (Boyce et al., 2014; Van Egdom et al., 2019; Nic Giolla Easpaig et al., 2020), an increase in time, e.g., due to the need to view a large number of results or poor usability (Aaronson et al., 1993; Cella et al., 1993; Boyce et al., 2014; Van Egdom et al., 2019; Nic Giolla Easpaig et al., 2020), and disruption of work routines (Boyce et al., 2014).

Questionnaires measuring HrQoL exist for a variety of oncological research areas. Developed for oncological clinical trials are the EORTC quality of life of cancer patients (EORTC QLQ-C30; Aaronson et al., 1993) and the Functional Assessment of Cancer Therapy-General (FACT-G; Cella et al., 1993). These and other comprehensive questionnaires often include a high number of items and are complex in their evaluation. Shorter HrQoL instruments often are generic (EQ-5D; Herdman et al., 2011), distress thermometer (DT; Mehnert et al., 2006) or miss cancer-specific dimensions (Robbeson et al., 2018). With the emerging barriers and facilitators in mind, there is a special need for a questionnaire allowing the perspicuous and efficient measurement of PROs in routine oncological care.

We have recently developed a short instrument measuring HrQoL in cancer patients, but it still needs psychometrical testing (Schrage et al., 2022). The instrument was developed using qualitative analysis of interviews and focus groups with patients and HCPs, and an expert discussion. It consisted of six dimensions (*Emotional Health, Physical Ailment, Autonomy, Social Functionality, Dignity, and Resources*) with all together nine items (see Table 1). The dimensions such as *Physical Ailment, Social Functionality*, and *Resources* had one item each. The dimensions such as *Emotional Health, Autonomy*, and *Dignity* each had more than one item (2–3). The response scaling of the items ranged from 0 (“not at all”) to 10 (“very much”) and was displayed as visual analog scales [comparable to the thermometer of the DT questionnaire (Mehnert et al., 2006)], in a horizontal format, with endpoints anchored verbally. According to the study protocol, we conducted an exploratory factor analysis, and the HELP questionnaire (“Hamburg Inventory for Measuring Quality of Life in Oncological Patients”) was implemented into routine oncological care. Information is gathered via a tablet with a direct transfer into the electronic patient record (Görlach et al., 2020). Patients are questioned at admission and discharge, as well as in between depending on the length of stay. At the same time as this study, the implementation was evaluated though the results of the implementation study will be displayed elsewhere.

**Table 1:** Dimensions and items of the recently developed HrQoL questionnaire.

**Dimensions**	**Item number**	**Items**
Emotional Health	1	How high is your emotional distress?
	2	How much do you worry about your illness?
	3	Do you feel sad or exhausted?
Physical Ailment	4	How severe are your physical problems?
Social Functionality	5	Do you feel sufficiently supported in your private life?
Autonomy	6	How severely are you affected by restrictions in your independence during your treatment?
		How much do you feel burdened by these restrictions?
Dignity	7	Are you being treated with respect during your treatment?
	8	Do you feel that medical staff see you as a person?
Resources	9	How capable do you feel of coping with your current situation?

To further develop the instrument, both exploratory and confirmatory tests are reasonable. Therefore, the primary aim of the present study is to evaluate and determine the psychometric properties of the developed HrQoL instrument (HELP) for use in the routine care of oncology patients with confirmatory analyses. In addition, we aimed to reduce the number of items to one item per dimension in order to develop a practical and short questionnaire.

## Materials and methods

The results presented here are part of the project “PRO-ONKO-Routine” (Schrage et al., 2020). The study protocol has been published elsewhere (Schrage et al., 2020). The project was funded by “Innovationsfond des Gemeinsamen Bundesausschusses” (funding number: 01VSF16024) and pre-registered at Open Science Framework (OSF)^1^. This study was reported in accordance with the STROBE statement (Von Elm et al., 2007).

### Study design

This observational study with a repeated measurements design with two-time points included oncological inpatients and outpatients from the University Medical Center Hamburg Eppendorf (UKE) in Germany. Patients were surveyed from five departments of the UKE: II. Medical Clinic and Polyclinic, Department of Stem Cell Transplantation, Department of Gynecology, Department of Radiotherapy and Radiation Oncology, and Department of Otolaryngology. All five departments are members of the University Cancer Center Hamburg. The study was approved by the local research ethic committee of the medical association Hamburg, no. PV5636. All participants provided written informed consent.

To evaluate the recently developed questionnaire for measuring HrQoL in cancer patients (Schrage et al., 2022), oncological inpatients and outpatients with different cancer entities were surveyed twice during the treatment. The questionnaire was presented to the participants in paper–pencil format.

A pilot run was conducted in May 2018. To assess the comprehensibility and feasibility of the questionnaire, three patients were instructed to use the think-aloud technique with concurrent verbalization to provide feedback on their understanding of the questionnaire (Wolcott and Lobczowski, 2021). In this way, the usability of the questionnaire, e.g., with regard to the comprehensibility of the items or the length of the questionnaire can be assessed and modified if needed. No changes in the questionnaire were necessary after this pilot run. Data collection started in June 2018 and ended in February 2019.

### Recruitment of participants

Inclusion criteria for patient recruitment were an age ≥ 18, a cancer diagnosis, sufficient language skills in German, and no severe cognitive or verbal impairments interfering with their ability to give informed consent. Potential inpatients to be questioned were pointed out by medical staff and addressed by scientific staff. In the outpatient departments, the oncological patients were addressed directly by scientific staff. For inpatients, questioning was conducted at the beginning of their cancer treatment and 3–7 days later. Cancer treatments included chemotherapy, radiation, surgery, or stem cell transplantation. Outpatients were asked to participate at one time during their cancer treatment and again 1 week later.

To be able to complete the recruitment in the time given, an incentive was introduced to study participants after the survey had been ongoing for 6 months. Every new participant was offered a drugstore voucher for 15,00€ when the addressed patient agreed to participate in the study.

### Measurements

For the evaluation of the questionnaire, a series of established standardized measurements were included in the quantitative survey at both points of measurement (see Supplementary material 1).

### Statistical analysis

**Figure 1:**
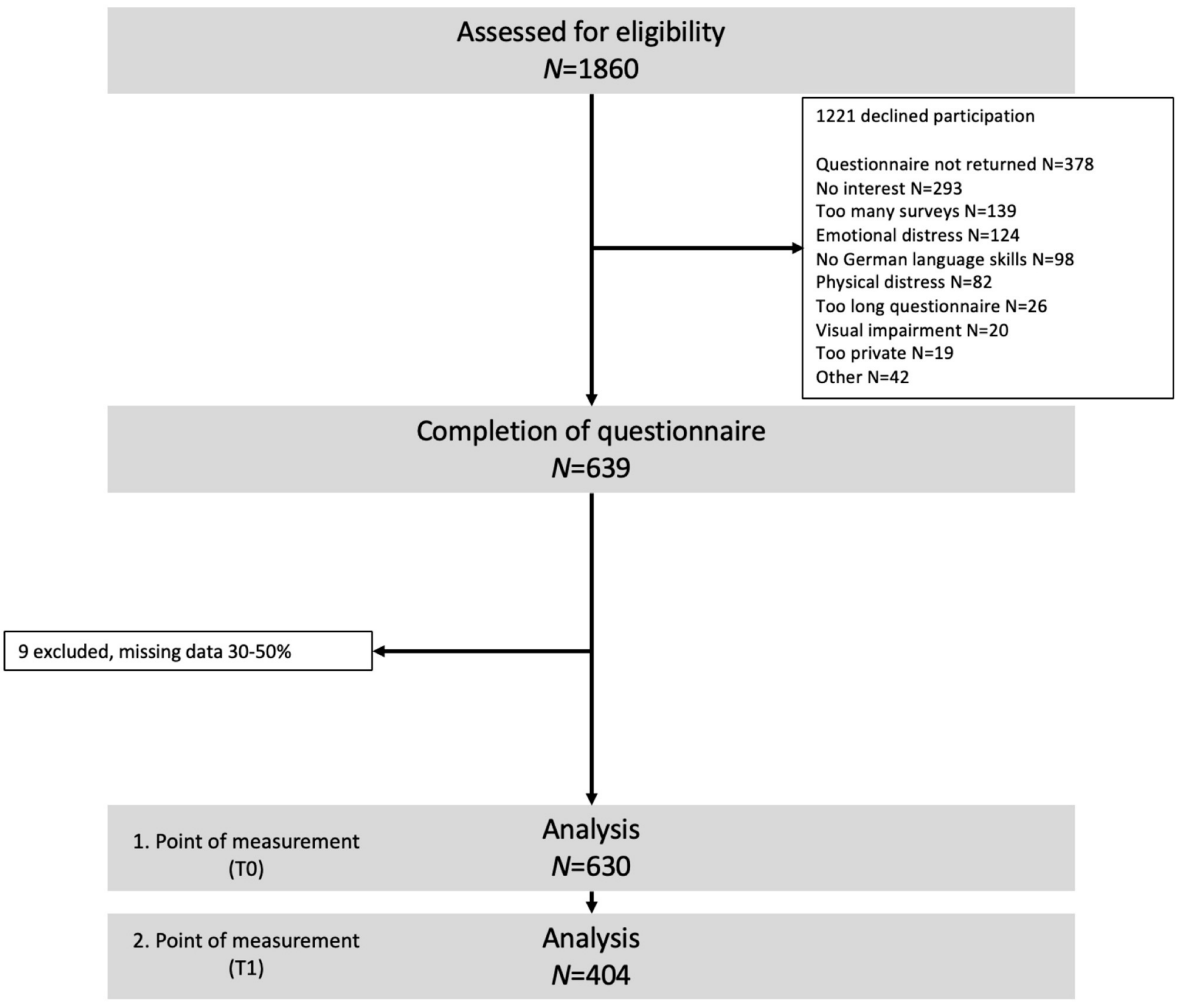
Flow diagram.

Descriptive statistics using frequencies for categorical data or mean values and standard deviation (*SD*) for interval-level data were computed to describe the sample. *N* = 9 cases had to be excluded from the analysis because of missing values > 30% (see Figure 1). For the confirmatory factor analysis, missing values were handled by maximum likelihood estimation. All other analyses were performed using complete cases only. We performed the validation and item reduction with data from the first point of measurement and the reliability and responsiveness to change analysis with data from both points of measurement.

On the basis of the previous qualitative study (Schrage et al., 2022), hierarchical confirmatory factor analyses (Greenhalgh et al., 2004) were conducted to test the factor structure of the instrument. The sample size is sufficient for this analysis (Mundfrom et al., 2005). Because of the skewed distribution in two items, the covariance matrix of the items was analyzed using robust maximum likelihood estimation. Parameters were standardized using the completely standardized solution where the variance of the factor (HrQoL) and the latent and observed variables are standardized. For the evaluation of the global model fit, we examined model fit indices with a cutoff of 0.95 for the Tucker–Lewis index (Mackler et al., 2017) and the Bentler comparative fit index (CFI) and a cutoff of below 0.08 for the root mean square error of approximation (RMSEA) and the standardized root mean square residual (SRMR) according to Hu and Bentler, in addition to the Akaike Information Criterion (AIC; Hu and Bentler, 1999). Two models were fitted: (1) a hierarchical model of the HELP with a general latent factor of HrQoL and with second-level latent factors of the six HELP dimensions resulting from previous qualitative analysis (*Emotional Health, Physical Ailment, Autonomy, Social Functionality, Dignity*, and *Resources*) and (2) an exploratory modified hierarchical model. This second model was fitted to further understand the comprehensive concept of HrQoL. We intended to examine with an additional exploratory model by adding a second level of latent factors, whether the model fit and factor loadings could improve and therefore have a better fit of the model. CFAs were conducted using the packages lavaan (Rosseel, 2012) and lavaanPlot (Alex, 2021) of the R Software (version 4.1.2) (The R Foundation, 2022). All further analyses were conducted using IBM SPSS Software (version 27).

The CFAs were conducted with nine out of 10 items, as presented in Table 1. The guiding principle for the development of the questionnaire was to represent each dimension with one item, which should usually be the item with the highest or in case of only a single item available a sufficient factor loading on the respective dimension. However, the item “How much do you feel burdened by these restrictions of your independence?” of the dimension *autonomy* refers to a previous item and therefore was excluded from the analyses. All inverse items were inverted to provide the same direction for every item.

Using the factor loadings as an indicator, the dimensions were reduced to one item each by choosing the item with the highest factor loading for each item. Convergent validity was tested by Pearson's product–moment correlations with established scales of standardized measurements assessing cancer-specific HrQoL, depressive and anxiety symptoms, and dignity. *A priori*, we specified that the HELP-6 dimension, *Emotional Health*, should be correlated with one of the two scales of the PHQ-4 (Lowe et al., 2010), the dimension *Physical Ailment* should be correlated with *Physical Wellbeing* of the FACT-G (Bonomi et al., 1996), *Autonomy* should be correlated with the scale *Loss of Autonomy* of the PDI-G (Sautier et al., 2014), *Social Functionality* should be correlated with the scale *Social Wellbeing* of the FACT-G (Bonomi et al., 1996), the dimension *Dignity* should be correlated with the scale *Loss of Sense and Worth and Meaning* of the PDI-G (Sautier et al., 2014), and *Resources* should be correlated with the scale *Functional Wellbeing* of the FACT-G (Bonomi et al., 1996). We defined a medium effect size, i.e., an *r* of ≥0.30, as a minimal critical value for a valid dimension of the newly developed questionnaire (Cohen, 1988).

Reliability was examined by test–retest reliability. Data from both points of measurement of the new one-item scales were correlated with each other (Pearson's product–moment correlation with bias-corrected and accelerated bootstrap 95% CIs).

Responsiveness to change was assessed by anchor-based determination of a minimal clinically important difference (MCID; Revicki et al., 2008) set to one *SD* of the change scores in related established PRO measures. The change scores were computed by subtracting the scores of the first point of measurement from the second. Thus, for each dimension of the developed questionnaire, we assigned one related dimension of a standardized PRO measure. This was done in the same procedure as for convergent validity, except for *Emotional Health* which was related to the DT. The patients were allocated to groups of “worsened,” “unchanged,” and “improved” determined by the MCID, and six one-way ANOVAs were used to determine the significance of a difference between the change groups.

A *p*-value of≤0.05 was considered significant (no correction for multiple testing), and we calculated *d* as the effect size measure (Cohen, 1988). With a sample of 404 patients, it is possible to detect a significant difference between groups with a small effect size of *f* = 0.15 with a power of 80% (Cohen, 1988).

## Results

### Description of sample

Of the *N* = 1,860 patients approached, data from *N* = 630 patients were included in the primary analyses. Participant flow is featured in Figure 1. The mean age was 59.2 years (*SD* = 13.63), with 56% being female participants. Most are employed (43%) or retired (34%), and nearly half had a low education. More than two-thirds (71%) are in a permanent relationship, live with a partner, parents, and or children (75%; Table 2). Half of the patients (52%, *n* = 329) were in distress (DT ≥ 5).

**Table 2:** Sample characteristics.

	**Total sample, first point of measurement**	**Second point of measurement**
	***N* = 630**	***N* = 404**
Age, mean (SD)	59.21 (13.63)	59.46 (13.58)
**Gender**		
Female	355 (56.3%)	219 (54.2%)
Male	275 (43.7%)	185 (45.8%)
**Education** ^a^		
Low	284 (45.1%)	187 (46.3%)
Medium	131 (20.8%)	80 (19.8%)
High	207 (32.9 %)	92 (32.9%)
Other	8 (1.3%)	4 (1.0%)
**Employment relationship**		
Employed	271 (43.1%)	171 (42.4%)
Self-employed	49 (7.8%)	25 (6.2%)
Apprentice/student	10 (1.6%)	6 (1.4%)
Homemaker	24 (3.8%)	15 (3.7%)
Unemployed	18 (2.9%)	12 (3.0%)
Retired	215 (34.1%)	153 (37.9%)
Incapacitated for work	43 (6.8%)	23 (5.7%)
Other	7 (1.1%)	0 (0.0%)
**Relationship status**		
Without permanent relationship	177 (28.1%)	108 (26.7%)
Married or in a permanent relationship	453 (71.9%)	296 (73.3%)
Number of children, mean (SD)	1.27 (1.05)	1.27 (1.11)
**Living situation**		
Alone	157 (24.9%)	100 (24.8%)
Living with partner/ children/parents	473 (75.1%)	302 (74.7%)
**Residence**		
Village	90 (14.3%)	57 (14.1%)
Small city (<25,000 residents)	87 (13.8%)	58 (14.4%)
Medium city (25,000–100,000 residents)	76 (12.1%)	53 (13.1%)
Large city (>100,000 residents)	377 (59.8%)	236 (58.4%)

^a^Low = graduation after 9 or 10 years of school, medium = graduation after 12 or 13 years of school, high = graduation after more than 13 years of school (e.g., colleague or university).

Cancer-related patient characteristics from electronic medical records could be retrieved from *n* = 479 (75.01%) patients. The most frequent cancer entities of this sub-sample were breast (125, 27.8%), prostate (47, 10.5%), and larynx (32, 7.1%). Approximately 72.4% (339) of the participants were outpatients. The patients were treated most often with chemotherapy (302, 63.0%) and/or surgery (300, 62.6%), further with radiotherapy (212, 46.0%), anti-hormonal therapy (85, 18.6%), and/or stem cell transplantation (10, 2.2%).

### Incentives

Because we had to change data collection procedures in the last 3 months of the accrual period by offering study participants incentives, we examined the data for imbalances in the sample with and without incentives. One hundred eighteen incentives were issued to patients who agreed to study participation, of these 72 filled out the questionnaire and were included in the analysis. We detected no imbalances between participants with (*n* = 72) and without (*n* = 558) incentives, using descriptive statistical analysis.

### Confirmatory factor analysis

**Figure 2:**
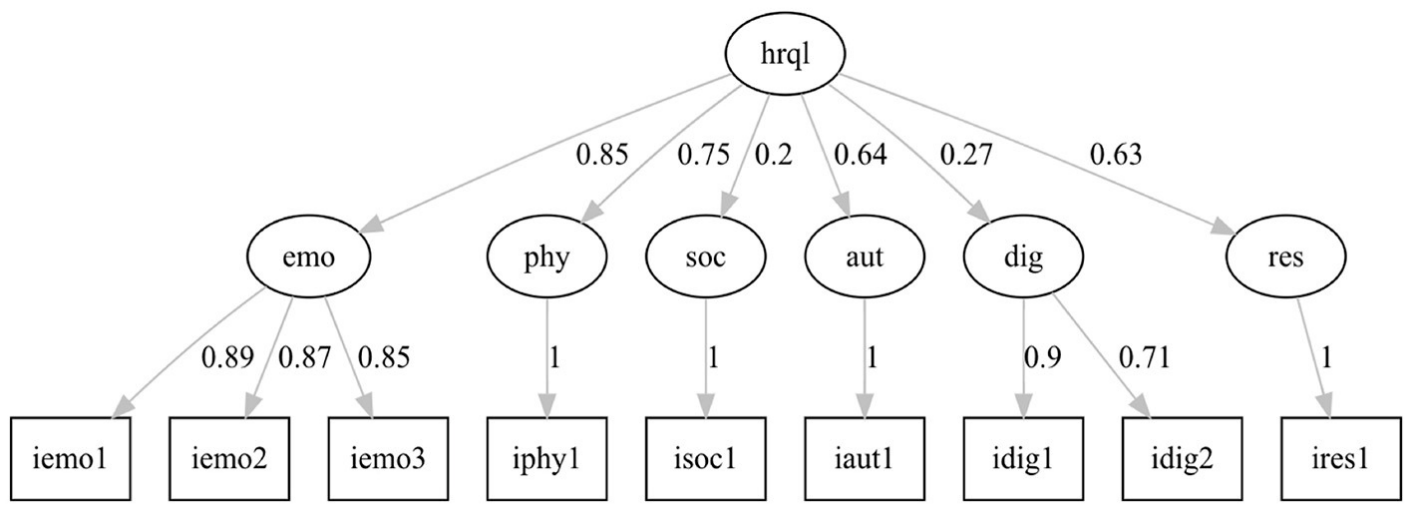
Hierarchical model of the HELP questionnaire, model 1.

**Figure 3:**
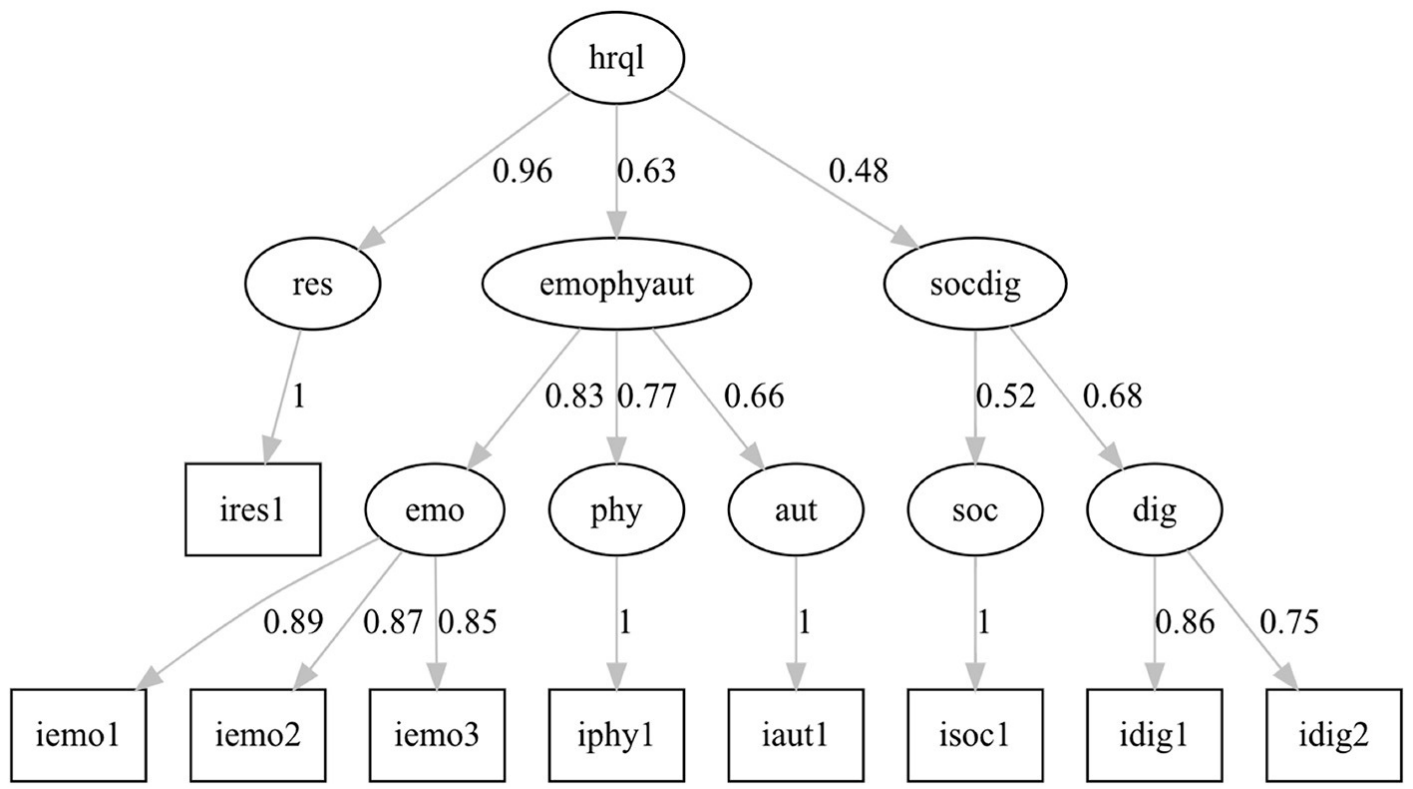
Modified hierarchical model of the HELP questionnaire, model 2.

A sufficiently large sample of *N* = 629 patients (Hair, 2014) could be included in the analysis. Model fit indices of the two models are presented in Table 3. Model 1 (Figure 2) showed a medium model fit with CFI = 0.93 and RMSEA = 0.10 not meeting the *a priori* set cutoffs. In contrast, model 2 (Figure 3) indicates a good model fit with a marginally lower AIC and fit indices SRMR and RMSEA below 0.08 and CFI above 0.95.

**Table 3:** Fit indices for the HELP questionnaire.

**Models**	***χ^2^***	***df***	***P***	**AIC**	**CFI**	**SRMR**	**RMSEA**
Hierarchical model (Model 1)	164.00	25	<0.001	23,226.0	0.93	0.07	0.10
Modified hierarchical model (Model 2)	107.00	23	<0.001	23,143.0	0.97	0.04	0.08

The factor loadings in model 1 of the latent factors representing the questionnaire dimensions on the general latent factor HrQoL are medium to high (0.64–0.85) except for the loadings of the factor *Social Functionality* (0.20) and *Dignity* (0.27). The standardized loadings of the manifest factors represent the questionnaire items ranging from 0.71 to 1.00. In model 2, a level of latent factors has been added to specify the relationship of the questionnaire dimensions to the overall HrQoL. One factor combines *Emotional Health, Physical Ailments*, and *Autonomy (emophyaut)*; a second factor combines *Social Functionality* and *Dignity (socdig)*; and a third factor represents the questionnaire dimension *Resources*. The factor loadings for these three latent factors range from 0.48 to 0.96. Higher factor loadings appear for the latent factors representing the six dimensions of the questionnaire loading on the combined factors emophyaut, socdig, and on HrQoL (0.52–0.96).

The second goal of this study was to reduce the number of items to one item per dimension. We based the selection of the items on the highest factor loadings from CFA. For the dimension *Emotional Health*, we selected item 1, for *Physical Ailments*, item 4 was selected, for *Autonomy*, item 6 was selected, for *Social Functionality*, item 3 was selected, for *Dignity*, item 7 was selected, and for *Resources*, item 9 was selected (Table 1). In the end, the new questionnaire entails six dimensions with one item each (Supplementary material 2).

### Reliability

Because the developed measurement entails single-item scales, we calculated the test–retest reliability of the first (T0) and the second (T1) measurement points with bivariate Pearson's product–moment correlations. The dimensions show good test–retest reliability with correlations ranging between 0.56 and 0.81 (*p* < 0.001; Table 4).

**Table 4:** Test–retest reliability of HELP-6.

	***N***	***r***	**95% CI**
		***p***	
Emotional Health	390	0.81	0.773–0.842
		<0.001	
Physical Ailments	390	0.74	0.691–0.782
		<0.001	
Autonomy	388	0.74	0.700–0.789
		<0.001	
Social Functionality	391	0.73	0.718–0.810
		<0.001	
Dignity	385	0.56	0.489–0.626
		<0.001	
Resources	387	0.72	0.672–0.767
		<0.001	

### Responsiveness to change

The change scores of the HELP-6 dimensions comparing patients with unchanged to patients with changed HrQoL over time differed significantly in three dimensions (Table 5). A significant difference with medium effect sizes between the three groups (worsened, unchanged, and improved) was found in the dimension *Emotional Health* with both planned contrasts being significant, in the dimension *Physical Ailments*, also with both planned contrasts being significant and a significant difference with the planned contrast worsened vs. unchanged in the dimension *Social Functionality*. In the other three dimensions, the three groups did not differ significantly.

**Table 5:** Responsiveness to change in HELP-6 dimensions.

**HELP-6 dimension**	**ANOVA**		**Planned contrast worsened vs. unchanged**				**Planned contrast improved vs. unchanged**		
	***F*** **(Mundfrom et al.**, **2005****)**	***p***	***n**_*w*_*	***n**_*u*_*	***p***	***d***	***n**_*i*_*	***p***	***d***
Emotional Health	*F*_(2, 380)_ = 12.64	<0.001	59	259	0.009	0.41	65	<0.001	0.53
Physical Ailments	*F*_(2, 342)_ = 31.14	<0.001	86	165	0.003	0.47	94	<0.001	0.74
Autonomy	*F*_(2, 375)_ = 0.10	0.901	16	343	0.974	0.01	19	0.648	0.11
Social Functionality	*F*_(2, 322)_ = 2.92	0.055	55	220	0.018	0.37	50	0.948	0.01
Dignity	*F*_(2, 343)_ = 0.66	0.516	84	196	0.270	−0.15	66	0.538	0.01
Resources	*F*_(2, 343)_ = 1.03	0.359	74	174	0.948	0.01	98	0.184	0.18

Anchor for Emotional Health was DT, for Physical Ailments was FACT-G Physical Wellbeing, for Autonomy was PDI-G Loss of Autonomy, for Social Functionality was FACT-G Social Wellbeing, for Dignity was PDI-G Loss of Sense and Worth and Meaning, and for Resources was FACT-G Functional Wellbeing.

### Convergent validity

To test whether the new questionnaire reflects the latent constructs of *Emotional Health, Physical Ailments, Autonomy, Social Functionality, Dignity*, and *Resources*, we tested the relations to standardized validated questionnaires and their dimensions by bivariate correlations. The correlations with bias-corrected and accelerated bootstrap 95% CIs are displayed in Table 6.

**Table 6:** Convergent validity—correlations of HELP with standardized questionnaires.

	**PHQ-4_d**	**FACT-G_pw**	**PDI-G_a**	**FACT-G_sw**	**PDI-G_swm**	**FACT-G_fw**
Emotional Health	**0.54** ^***^ **[0.479, 0.592]**	−0.51^***^ [−0.572, −0.451]	0.20^**^ [0.121, 0.273]	−0.19^***^ [−0.266, −0.105]	0.44^***^ [0.368, 0.499]	−0.55^***^ [−0.603, −0.489]
Physical Ailments	0.47^***^ [0.408, 0.531]	−0.68^***^[−0.722, **−0.633]**	0.37^***^ [0.303, 0.439]	−0.09^*^ [−0.175, −0.010]	0.40^***^ [0.332, 0.468]	−0.53^***^ [−0.589, −0.472]
Autonomy	0.41^***^ [0.339, 0.471]	−0.55^***^ [−0.604, −0.489]	**0.36** ^***^ **[0.285, 0.423]**	−0.04 [−0.123, 0.044]	0.33^***^ [0.251, 0.396]	−0.55^***^ [−0.604, −0.489]
Social Functionality	−0.21^***^ [−0.286, −0.135]	0.17^***^ [0.090, 0.249]	−0.09 [−0.168, −0.012]	**0.48** ^***^ **[0.411, 0.540]**	−0.28^***^ [−0.353, −0.204]	0.19^***^ [0.111, 0.268]
Dignity	−0.18^***^ [−0.258, −0.106]	0.22^***^ [0.141, 0.297]	−0.07 [−0.145, 0.013]	0.26^***^ [0.182, 0.338]	−0.25^***^[−0.324, **−0.172]**	0.24^**^ [0.163, 0.318]
Resources	−0.51^***^ [−0.568, −0.452]	0.51^***^ [0.451, 0.572]	−0.32^***^ [−0.386, −0.244]	0.20^***^ [0.117, 0.277]	−0.51^***^ [−0.563, −0.442]	**0.52** ^***^ **[0.458, 0.577]**

ns, not significant (*p* > 0.05).

^*^p < 0.05, ^**^p < 0.01, ^***^p < 0.001, BCa bootstrap 95% CIs reported in brackets, n = 551–621, sample sizes for every correlation reported in Supplementary material 3.

PHQ-4_d, depressive symptoms, PHQ-4; FACT-G_pw, Physical Wellbeing, FACT-G; PDI-G_a, Loss of Autonomy, PDI-G; FACT-G_sw, Social Wellbeing, FACT-G; PDI-G_swm, Loss of Sense and Worth and Meaning, and PDI-G; FACT-G_fw, Functional Wellbeing, FACT-G. These values are highlighted in bold because they represent the correlations between the scales of the new questionnaire and those of the standardized questionnaires. The other values of the correlations are also listed in the table for reasons of transparency.

The item “How high is your emotional distress?” of the dimension *Emotional Health* correlated highly (*r* = 0.54) with the depression dimension of the PHQ-4 (Lowe et al., 2010). Highly correlated with each other were the dimensions *Physical Ailments* (item: “How severe are your physical problems?”) and *Physical Wellbeing* of the FACT-G with *r* = −0.68, *Social Functionality* (item: “Do you feel sufficiently supported in your private life?”) and *Social Wellbeing* of the FACT-G (Cella et al., 1993) with *r* = 0.48, and the dimension *Resources* (item: “How capable do you feel of coping with your current situation?”) with the dimension *Functional Wellbeing* of the FACT-G, *r* = 0.52. Two dimensions, *Dignity* (*r* = 0.25, item: “Do you feel that medical staff see you as a person?”) and *Autonomy* (*r* = 0.36, item: “How severely are you affected by restrictions in your independence during your treatment?”) of the new questionnaire correlated moderate to low with the standardized dimensions of the PDI-G (Sautier et al., 2014).

The questionnaire developed in this way was named “HELP-6” (“Hamburg Inventory for Measuring Quality of Life in Oncological Patients-6”) and is visualized in Supplementary material 2.

## Discussion

With the present study, we aimed to evaluate the HrQoL measurement for use in oncological patients in the clinical routine developed in a previous study (Schrage et al., 2022). The secondary aim was the reduction to one item per dimension. The results revealed a measurement with six dimensions (*Emotional Health, Physical Ailment, Autonomy, Social Functionality, Dignity*, and *Resources*) and one item for each dimension, named the Hamburg Inventory for Measuring Quality of Life in Oncological Patients-6 (HELP-6).

We used confirmatory factor analysis to test the results from the previous qualitative analysis of our data structure. With a hierarchical approach, we fitted a model with moderate to good model fit. To test whether the fit of a model can be improved, we exploratively modified the first model. An additional factor level, loading on the general factor HrQoL, was included to better map the relationship of the dimensions to each other and to HrQoL. *Emotional Health, Physical Ailment*, and *Autonomy* were combined in one factor because of their high association with each other. *Resources* was left as a single factor because content-wise it does not combine well with the other dimensions. At last, *Social Functionality* and *Dignity* were combined in one factor. Both factors had low factor loadings in the first model. Additionally, both relate to how other people treat the questioned patient (in a social or clinical environment). Chochinov et al. (2002) even assessed that one of the three major categories of dignity is the “social dignity inventory” which entails issues such as privacy boundaries and social support. Our modified model with the added factor level showed an improved model fit and even improved factor loadings, especially for *Social Functionality* and *Dignity*. In this second model, *Resources* is presented with a very high factor loading of 0.96. This implies that *Resources* is a strong factor to HrQoL, contrary to the rare occurrence in other HrQoL instruments. Additionally, it serves the necessity often called for to determine the need for support in patients and therefore being able to infer actions by clinicians (Anatchkova et al., 2018; Nguyen et al., 2021; Schrage et al., 2022). The additional latent factor level in the modified model still entails the original six latent factors, which are directly connected to the according items. Both models show that it might be clinically relevant to assume the six dimensions as different and to be able to respond to them in a differentiated way. As the second model is an explorative approach, it needs to be tested and confirmed in other studies.

Responsiveness to change between groups (worsened, unchanged, and improved) was confirmed only in three dimensions: *Emotional Health, Physical Ailments, and Social Functionality*. It could not be found for the dimensions *Autonomy, Dignity*, and *Resources*. This measurement was developed with the intended use of monitoring with a higher frequency, to detect and react to changes in a patient's HrQoL outcomes. Regarding the anchors that were selected, the DT and Fact-G are sensitive to change, though nothing is yet known about the changing responsiveness of the PDI (used as an anchor for the dimensions *Autonomy* and *Dignity*). However, the PDI is so far the only standardized questionnaire for measuring dignity in German (Sautier et al., 2014). Furthermore, it is in question whether the scale *Functional Wellbeing* is a fitting anchor for the dimension *Resources*. The scale resources asks after a patient's perceibed capability to cope with her or his situation, and the scale functional well-being asks after the capability to work, sleep well, and enjoy free time, which is not the same. According to our preceding study, the ability to cope is an important part of HrQoL and is seldom included in HrQoL measurement, which is why we included this dimension in the HELP-6. Though assessing the responsiveness to change for the dimension *Resources* should be repeated with a different anchor. Altogether, the results from this analysis might be compromised by a wide range of the sample size and partly skewed distribution. In most comparisons, the groups were unequal and/or had a small sample size. This holds especially for the groups built for the dimension *Autonomy*. Thus, it remains important in future studies to reassess the change sensitivity of the HELP-6 scales.

In a final step, we tested the convergent validity of the HELP-6 dimensions by correlating them with dimensions of standardized measurements. Four dimensions reached a high correlation though two dimensions (*Autonomy* and *Dignity*) correlated only moderate to low. Both dimensions were correlated with the dimensions of the PDI-G (Sautier et al., 2014). The two respective PDI-G dimensions are not specified in a hospital setting or treatment situation. We assume this could be the reason that the two HELP-6 dimensions did not correlate higher, as they explicitly inquire after being treated with respect during their treatment (*Dignity*) and the limitation to their independence during treatment (*Autonomy*). This seems to be a distinction worth noticing and is compliant with other studies which state the importance of the intended setting for the use of PROMs (Samuel et al., 2019; Schrage et al., 2022). Otherwise, the dimensions are not distinct and have high intercorrelations with other dimensions of the questionnaire.

### Strengths and limitations

To be able to complete the recruitment in the time given, we had to change the study design during data collection. We offered incentives to patients questioned in the last 3 months of the ongoing survey. Our analyses show that participants with incentives did not differ at baseline from participants without incentives, which is why we included the group with incentives in the analyses. Another limitation is that we might not have been able to include patients in high distress. A total of 208 addressed patients declined participation due to mental or physical distress. Nonetheless, the results of the DT depict that more than half of the participants in our sample were in distress (≥5). Furthermore, the results from the responsiveness of change analysis need to be re-assessed in future studies because of unequal groups and partly a small sample size.

Lastly, a few patients from the department of stem cell transplantation could be included, which explains the small number of patients in our sample treated with transplantation.

The strengths of this study are next to a large sample size that we conducted the study at a certified Comprehensive Cancer Center. Thus, we were able to include inpatients and outpatients with various cancer entities and treatment stages. This increases the generalizability of the entire routine oncological care. Another strength is the inclusion of standardized, validated, and commonly used instruments for validation. This strengthens the quality of validation of the HELP-6.

### Implications for practice and conclusion

We conclude that with the HELP-6 instrument for measuring HrQoL in cancer patients, we provide a short and practical PROM. Assuming less time is required for both patient and clinician, the instrument is still informative on important HrQoL dimensions. The HELP-6 could even allow clinicians to determine if the patient needs further psycho-oncology treatment or the assistance of a social worker. Especially due to the increased administration of oral therapy, this short instrument could be useful for monitoring patients' HrQoL. All in all, the instrument could be an important addition to inpatient and outpatient cancer care.

For future research, the HELP-6 should be evaluated in a multicenter study to assess its generalizability. It should be further examined regarding responsiveness to change, usability, and feasibility in different inpatient and outpatient settings (e.g., smaller clinics and primary care facilities) and the clinical benefit of the instrument should be assessed. The measurement was designed to be of use to patients and clinicians, which is important in every routine care setting. As another next step, it is also interesting to assess whether the HELP-6 is applicable to other diseases.

## Data availability statement

The raw data supporting the conclusions of this article will be made available by the authors, without undue reservation.

## Ethics statement

The studies involving human participants were reviewed and approved by Ethics Committee of the Physician's Chamber in Hamburg, Weidestr. 122 b, 22083 Hamburg. The patients/participants provided their written informed consent to participate in this study.

## Author contributions

Material preparation and data collection were performed by MG and TS. Analysis was performed by TS and HS. The first draft of the manuscript was written by TS. All authors contributed to the study's conception, design, commented on previous versions of the manuscript, read, and approved the final manuscript.

## Footnotes

^1^OSF - Open Science Framework [Online]. Available: https://osf.io/ [Accessed February 2020].
