# Supplementary material for: Evaluation of a short instrument for measuring health-related quality of life in oncological patients in routine care (HELP-6): an observational study
Source: Front Psychol. 2023 May 16;14:1158449. doi: 10.3389/fpsyg.2023.1158449 (PMC10228503; doi:10.3389/fpsyg.2023.1158449)
Supplement: Supplementary file 1 [file Data_Sheet_1.docx]

**Supplementary Information S1**

Study Design – Measurements

To assess HrQoL in cancer patients we included the Functional Assessment of Cancer Therapy – General (FACT-G) (Bonomi et al., 1996). It measures four dimensions of well-being (physical, social, emotional, and functional) with 27 five-point Likert-type items. Subscales range from 0 to 28 (0-24 for emotional well-being) and the total score from 0-108, with higher scores indicating better quality of life (Bonomi et al., 1996). Additionally, the distress scale of the Distress-Thermometer (DT) (Mehnert et al., 2006) often used in oncological practice was included. The distress scale measures distress from 0 (“no distress”) to 10 (“extreme distress”). The cut-off for the German population is ≥5 (Mehnert et al., 2006).

Further, to assess depressive and anxiety symptoms, we used the Patient Health Questionnaire 4 (PHQ-4) (Lowe et al., 2010). This short questionnaire has one dimension each on depressive and anxiety symptoms (Lowe et al., 2010). The higher the sum score, the worse are the according symptoms, whereby the score can be built for the subscales or as a total score (Lowe et al., 2010). Dignity was assessed by using the Assessment of Patients’ Dignity in Cancer Dare (PDI-G) (Sautier et al., 2014). With 25 items and four dimensions (Loss of Sense of Wort and Meaning, Anxiety and Uncertainty, Physical Symptom Distress and Body Image, Loss of Autonomy) the PDI-G measures the most common factors influencing a persons’ sense of dignity. A high score represents a greater problem regarding factors of dignity (Sautier et al., 2014).

The sociodemographic questionnaire asked for age, gender, education, employment relationship, family status, children and living situation. Also, the 10 newly developed items (see Table 1) were part of the survey to be able to analyze their psychometric properties. Since the technical equipment was not ready for use in all departments, this survey was presented to the patients in paper-pencil format. Medical data (cancer entity, type of treatment, and treatment as in- or outpatient regarding the current treated cancer) were retrieved from the electronic patient record. However, it was not possible to retrieve data from all participants.
