# Supplementary material for: Evaluation of a short instrument for measuring health-related quality of life in oncological patients in routine care (HELP-6): an observational study
Source: Front Psychol. 2023 May 16;14:1158449. doi: 10.3389/fpsyg.2023.1158449 (PMC10228503; doi:10.3389/fpsyg.2023.1158449)

**Supplementary Information S2**

**HELP-6**

Cancer can impose various stresses and restrictions on your quality of life. Please use the enclosed scale to rate how stressed or restricted you currently feel:

**How high is your emotional distress?**


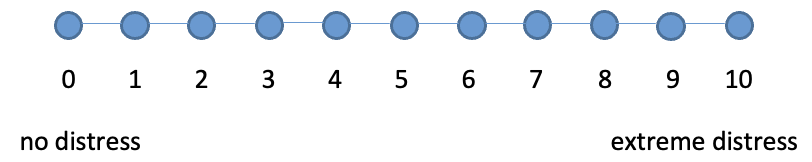


**How severe are your physical problems?**


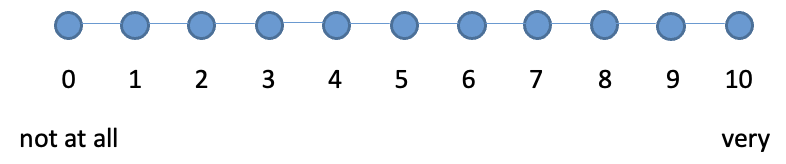


**Do you feel sufficiently supported in your private life?**


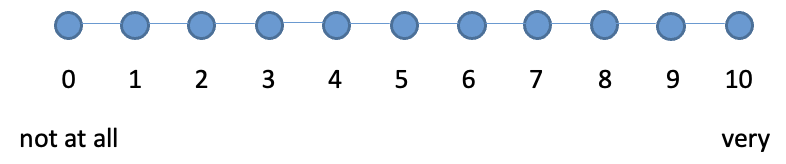


**How severely are you affected by restrictions in your independence during your treatment?**


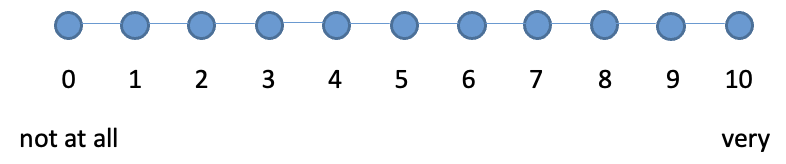


**Are you being treated with respect during your treatment?**


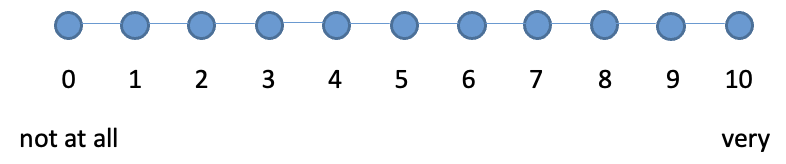


**How capable do you feel of coping with your current situation?**


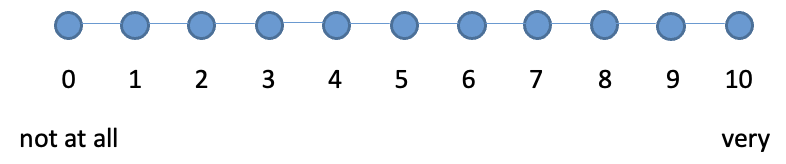

Supplement: Supplementary file 2 [file Data_Sheet_2.docx]
