# Supplementary material for: Evaluation of a short instrument for measuring health-related quality of life in oncological patients in routine care (HELP-6): an observational study
Source: Front Psychol. 2023 May 16;14:1158449. doi: 10.3389/fpsyg.2023.1158449 (PMC10228503; doi:10.3389/fpsyg.2023.1158449)
Supplement: Supplementary file 3 [file Data_Sheet_3.docx]

**Supplementary Information S3**

*S3: Correlations of HELP dimensions with dimensions of standardized questionnaires including sample size*

|  | PHQ-4_d | FACT-G_pw | PDI-G_a | FACT-G_sw | PDI-G_swm | FACT-G_fw |
| --- | --- | --- | --- | --- | --- | --- |
| Emotional health | **.54*****  **[.479, .592]**  **619** | -.51***  [-.572, -.451]  574 | .20**  [.121, .273]  615 | -.19***  [-.266, -.105]  552 | .44***  [.368, .499]  586 | -.55***  [-.603, -.489]  576 |
| Physical ailments | .47***  [.408, .531]  620 | **-.68*****  **[-.722, -.633]**  **574** | .37***  [.303, .439]  615 | -.09*  [-.175, -.010]  552 | .40***  [.332, .468]  586 | -.53***  [-.589, -.472] 576 |
| Autonomy | .41***  [.339, .471]  617 | -.55***  [-.604, -.489] 573 | **.36*****  **[.285, .423]**  **613** | -.04  [-.123, .044]  552 | .33***  [.251, .396]  584 | -.55***  [-.604, -.489]  574 |
| Social functionality | -.21***  [-.286, -.135]  621 | .17***  [.090, .249] 576 | -.09  [-.168, -.012]  617 | **.48*****  **[.411, .540]**  **554** | -.28***  [-.353, -.204]  588 | .19***  [.111, .268]  578 |
| Dignity | -.18***  [-.258, -.106]  615 | .22***  [.141, .297] 572 | -.07  [-.145, .013]  610 | .26***  [.182, .338]  551 | **-.25*****  **[-.324, -.172]**  **582** | .24**  [.163, .318]  572 |
| Resources | -.51***  [-.568, -.452]  618 | .51***  [.451, .572]  574 | -.32***  [-.386, -.244]  613 | .20***  [.117, .277]  553 | -.51***  [-.563, -.442]  585 | **.52*****  **[.458, .577]**  **574** |
